# 

**Published:** 1918-02-09

**Authors:** 


### CONTENTS.

PAGE

**Words and Things** ... ... ... ... ... ... 385

**War Office Increases Payments to Hospitals** 386

**Hospital and Institutional News** ... ... ... 387

A Chair of Preventive Medicine—The Memorial Buildings at Leeds—The New Supervision of Orthopædic Cases—A Big Proposal at Bradford—"P Stands for ——"—The Porthkerry Bed—An Officer's Munificence—An Argument to Annual Subscribers—Hospital Saturday Fund Grants—Card Games in Convalescence—Three Authorities Powerless Before an Epidemic—Working Under Difficulties—The Buried Side of Hospital Life—After-care of Consumptives—Hospital Employee Charged—Hospital for Tuberculous Children—The Problem of Depleted Staffs—The Retention of a Secretary—Men for the V.T.C.—The late Professor Remington—This Week's Drug Market.

**The Great War** ... ... ... ... ... ... ... 391

Wonderful Progress in Evacuation and Care of the Wounded.

**Lord Lister** ... ... ... ... ... ... ... 393

III. Thirty Momentous Years.

**Parliament and the Profession** ... ... ... 394

**Some Interesting Eye Conditions** ... ... 395

Unrecognised Unilateral Blindness.

**Territorial Medical Corps** ... ... ... ... 396

Promotion of Officers.

PAGE

**A Notable General Practitioner** ... ... ... 397

Staff-Surgeon Colonel John Aikman, M.D., C.M.

**Henry Maudsley, M.D., F.R.C.P.** ... ... 397

A Champion on the Side of Science.

**Gifts and Bequests** ... ... ... ... ... ... 398

**The Editor's Letter-Box** ... ... ... ... ... 399

The Voluntary Hospitals—Nerve-shattered Pensioners: More "Country Hosts" Needed—Mr. Smallwood's Complaint—Mission Hospitals and Publicity.

**Nursing Affairs in Ireland** ... ... ... ... 400

The Midwives Bill.

**The Matrons' and Sisters' Department** ... 401

The Growth of the Nursing Profession: III. The Work of Reorganisation.

Round the Hospitals.

Trained Nurses Support Nation's Tribute—Commonsense and Justice Conquer—Hospital Chairmen's Responsibility—Probationers Must be Trained—Existing Injustices Condemned—War Must not Stop Training.

**Compensation and Aid for Air-Raid Victims** 405

**Passing Events and Topics** ... ... ... ... 405

**Matrons' and Sisters' Appointments** ... ... 406

**The Book Market** ... ... ... ... ... ... 406

**The Institutional Worker** ... ... (Suppl.) 1

A System of Timekeeping for the Nursing and Domestic Staff: Answer to December Question.

